# Root extractive from *Daphne genkwa* benefits in wound healing of anal fistula through up-regulation of collagen genes in human skin fibroblasts

**DOI:** 10.1042/BSR20170182

**Published:** 2017-04-28

**Authors:** Dong Yang, Jun-hua Xu, Ren-jie Shi

**Affiliations:** 1 First Clinical Medical College, Nanjing University of Chinese Medicine, Nanjing, 210023, P.R. China; 2 Department of Anorectal Surgery, The First People’s Hospital of Lianyungang, Lianyungang 222002, P.R. China; 3 Department of Anorectal Surgery, Affiliated Hospital of Nanjing Uiversity of Chinese Medicine, Nanjing 210029, P.R. China; 4 Department of Anorectal Surgery, Jiangsu Province Hospital of Traditional Chinese Medicine, Nanjing 210029, P.R. China

**Keywords:** Anal fistula, Cell signaling pathways, Daphne genkwa, human skin fibroblasts, Wounds healing

## Abstract

Wound healing is the main problem in the therapy of anal fistula (AF). *Daphne genkwa* root has been traditionally used as an agent to soak sutures in operation of AF patients, but its function in wound healing remains largely unclear. The aim of the present study was to illuminate mechanisms of *D. genkwa* root treatment on AF. In the present study, 60 AF patients after surgery were randomly divided into two groups, external applied with or without the *D. genkwa* extractive. Wound healing times were compared and granulation tissues were collected. *In vitro*, we constructed damaged human skin fibroblasts (HSFs) with the treatment of *TNF-α* (10 μg/ml). Cell Count Kit-8 (CCK-8) and flow cytometry analysis were used to determine the effects of *D. genkwa* root extractive on cell viability, cell cycle and apoptosis of damaged HSFs. Furthermore, protein levels of *TGF-β, COL1A1, COL3A1, Timp-1*, matrix metalloproteinase (MMP)-3 (*MMP-3*) and *MEK/ERK* signalling pathways were investigated both *in vivo* and *in vitro*. Results showed that *D. genkwa* root extractive greatly shortens the wound healing time in AF patients. In granulation tissues and HSFs, treatment with the extractive significantly elevated the expressions of *COL1A1, COL3A1, Timp-1, c-fos* and *Cyclin D1*, while reduced the expression of *MMP-3*. Further detection presented that *MEK/ERK* signalling was activated after the stimulation of extractive in HSFs. Our study demonstrated that extractive from *D. genkwa* root could effectively improve wound healing in patients with AF via the up-regulation of fibroblast proliferation and expressions of *COL1A1* and *COL3A1*.

## Introduction

Anal fistula (AF) is an anorectum disease characterized as an abnormal communication between the anal glands and the perianal skin [1], which could cause leakage, discomfort and occasional pain in patients [2]. This disease mostly occurs in male patients and can be ascribed to excessive secretion of sex hormone [3]. Currently, surgery is the standard treatment, but it remains controversial to be an effective therapy for some potential risk factors, such as internal open wound, prolonged wound healing and susceptible infection [4–6]. Therefore, it is of great necessity to shorten the wound healing time and repair contaminated wounds after operation.

It has been reported that the basic pathology and physiology of wound healing includes three stages, including local inflammation response, cell proliferation and granulation tissue formation and tissue reconstruction, which determined the time and quality of wound healing [7]. The complex healing process consisted of interaction of structural protein, growth factors and protein kinase, as well as consequence of repaired cell proliferation, differentiation and apoptosis etc. [8]. Among these, granulation tissues played a crucial role in the process of wound healing. As the main cellular constituents of granulation tissues, fibroblast cells could regulate reparative process by secreting a variety of types of cytokines [9].

*Daphne genkwa* is a medicinal plant widely distributed in Yellow and Yangtze Rivers regions in China, which has already been used as traditional agent for anti-inflammation, tranquilization, analgesia and anticonvulsion [10]. It has been demonstrated that the root of *D. genkwa* constituted the majority of the secondary metabolites including flavonoids, coumarins and diterpenoids [11]. Previous studies presented that total flavonoids from the root of *D. genkwa* possessed profiles of anti-inflammatory [10], immunomodulatory [12], analgesic [13] and even antitumour activities [14]. However, the function of *D. genkwa* root on wound healing after operation in patients with AF remains unclear. Thus, the potential mechanism and activity of *D. genkwa* root need to be ascertained.

In the present study, we investigated the effects of *D. genkwa* root on cell proliferation, cell cycle and apoptosis of human skin fibroblasts (HSFs), as well as the mechanism underlying the biological functions. These findings might provide a meaningful basis for clinical application of *D. genkwa* on tissues repair of contaminated wound healing in patients with AF.

## Materials and methods

### Plant materials and extraction

*D. genkwa* was obtained from Nanjing University of Chinese Medicine. The roots of *D. genkwa* (5 kg) were well air-dried, chopped and extracted with double distilled water, as described previously [15]. The obtained root extractive from *D. genkwa* was used in the treatment of following tissues and cell lines.

### Granulation tissue collection

Total 60 patients with AF (from 2015 to 2016) were enrolled from the Department of Anorectal Surgery in the First People’s Hospital of Lianyungang and randomly divided into two groups: treatment group and control group. All the patients accepted therapy of cutting with thread ligation, but for *D. genkwa* treated group, the sutures were soaked in *D. genkwa* root extractive (30 min, 90°C) and after operation, the extractive was smeared on the wound once a day (1 week). PBS was used in control group correspondingly. Informed written consent was taken from every patient. The present study was reviewed and approved by medical ethics committee of Affiliated Hospital of Nanjing University of Chinese Medicine. After 7 days, fresh granulation tissues were obtained from the surface of the wound in these patients. A part of granulation tissues were stored in liquid nitrogen for quantitative real-time PCR (qRT-PCR) and Western blotting. The other resected specimens were fixed in 10% formalin solution and embedded in paraffin for immunohistochemistry assay.

### Cell lines and cell treatment

Cheloid HSFs (c-HSFs) and HSFs were purchased from the cell bank of Chinese Academy of Science, Shanghai. The c-HSFs were separated from cheloid. All cells were cultured in DMEM medium containing 20% FBS, 100 U/ml penicillin and 100 μg/ml streptomycin. Then, c-HSF cells were treated with 1, 5, 10, 25, 50 and 100 μg/ml of root extractive from *D. genkwa*. HSF cells were added with TNF-α (10 ng/ml) to build damaged cell model and also for the treatment by root extractive from *D. genkwa*. These cells were incubated at 37°C in humidified atmosphere of 5% CO_2_.

### qRT-PCR assay

Total RNA from tissues and cells after 48 h cultivation was extracted using TRIzol reagent (Invitrogen, Carlsbad, CA, U.S.A.) according to the manufacturer’s instructions. cDNA was synthesized from 1 μg of total RNA by using the Bestar qPCR RT Kit (DBI Bioscience, Ludwigshafen, Germany). Real-time PCR was performed on Stratagene Mx3000P Real time PCR platform (Agilent Technologies, New Castle, DE, U.S.A.) using total volume of 20 μl DBI Bestar® SybrGreen qPCR master Mix (DBI Bioscience, Ludwigshafen, Germany). The *Β-actin* was used as an internal control. Each sample was run in triplicate in three independent experiments. Relative quantification was determined by the method of 2^−ΔΔ*C*^_t_. The primer sequences were as follows:* COL1A1* (forward: 5′-GACGAAGACATCCCACCAATC-3′ and reverse: 5′-GGAGACCACGAGGACCAGAG-3′), *COL3A1* (forward: 5′-GCTGGCATCAAAGGACATCG-3′ and reverse: 5′-CAACACCACCACAGCAAGGA-3′), *TIMP-1* (forward: 5′-GGGGACACCAGAAGTCAACC-3′ and reverse: 5′-GCATTCCTCACAGCCAACAG-3′), matrix metalloproteinase (MMP)-3 (*MMP-3*) (forward: 5′-CCCTGATGTCCTCGTGGTA-3′ and reverse: 5′-GGTCCTGAGAGATTTTCGC-3′), *Cyclin D1* (forward: 5′-CCCTCGGTGTCCTACTTC-3′ and reverse: 5′-TTTGCGGATGATCTGTTTGT-3′) and *c-fos* (forward: 5′-CCGAAGGGAAAGGAATAAGA-3′ and reverse: 5′-TGCTGGGAACAGGAAGTCA-3′).

### Western blot assay

Total proteins were extracted from tissues and cells after 72 h cultivation by using lysis buffer (20 mM Tris/HCl, pH 7.4, 150 mM NaCl, 1 mM EDTA) and quantified by Pierce BCA Protein Assay Reagent Kit (Thermo Fisher Scientific, Waltham, MA, U.S.A.) according to the manufacturer’s protocol. Equal amount of protein was separated on denaturing SDS gel and transferred to a PVDF membrane. The membrane was blocked with 10% skim milk and the incubated with primary antibodies overnight at 4°C, followed by incubation in appropriate horseradish peroxidase (HRP)–conjugated secondary antibody (Santa Cruz Biotechnology, Santa Cruz, CA, U.S.A.). The membrane was washed twice with PBS and blots were then visualized by ECL system (Bio–Rad Laboratories, Hercules, CA, U.S.A.). *GAPDH* was used as loading control. The specific primary antibodies were as follows: anti-*COL1A1, COL3A1, Timp-1, MMP-3, Cyclin D1, c-fos* (Abcam, U.S.A.), *MEK1/2* and *p-MEK1/2, ERK1/2* and *p-ERK1/2* (Cell Signaling, Beverly, MA, U.S.A.).

### Immunohistochemistry

In brief, the sections from paraffin-embedded tissue were deparaffinized in xylene, rehydrated in ethanol, washed in PBS and blocked using 5% goat serum. Then, these sections were incubated with anti-*COL1A1, COL3A1* and *ERK1/2* (Santa Cruz Biotechnology, Santa Cruz, CA, U.S.A.) at 4°C overnight, followed by incubation with HRP–conjugated secondary antibody for 1 h at room temperature. Immunoreactivity was detected with the DAB kit (Vector Laboratories, Burlingame, CA, U.S.A.) and counterstained slightly with haematoxylin. Section images were captured using a microscope (Nikon, Chiyoda, Japan).

### CCK-8 assay

Cell Count Kit-8 (CCK-8, Beyotime, Beijing, China) was used to measure the number of viable cells from different treatments. Briefly, cells were digested, resuspended and reseeded into 96-well plates. Then, 10% CCK-8 solution was added into each well. After 1-h incubation, the absorbance was determined on ELISA reader (Bio–Rad, Hercules, CA, U.S.A.) at a wavelength of 450 nm.

### Flow cytometry analysis for cell cycle and apoptosis

Cells from different treatments were collected and seeded on 6-cm dishes at a density of 1 × 10^5^ cells/dish. For cell-cycle analysis, cells were added in 0.7 ml of 70% ethanol and then stained by adding propidium iodide (PI) solution, followed by incubation for 30 min at room temperature. The suspension was filtered through a 50-mm nylon mesh and stained cells were analysed a FACSCalibur flow cytometer (BD, New Jersey, U.S.A.). For cell apoptosis, it was detected by Annexin V/PI apoptosis detection kit (MULTI SCIENCES, Hangzhou, Zhejiang, China) following manufacturer’s instructions.

### Statistical analysis

The differences between groups were compared using Student’s *t* test and quantificative data were expressed as means ± S.D. of three independent experiments. Statistically significant difference was accepted at *P*<0.05.

## Results

### *D. genkwa* promoted wound healing and led to the expression change of COL1A1, COL3A1, TIMP-1, MMP-3, TGF-β, MEK1/2 and ERK1/2

**Figure 1:**
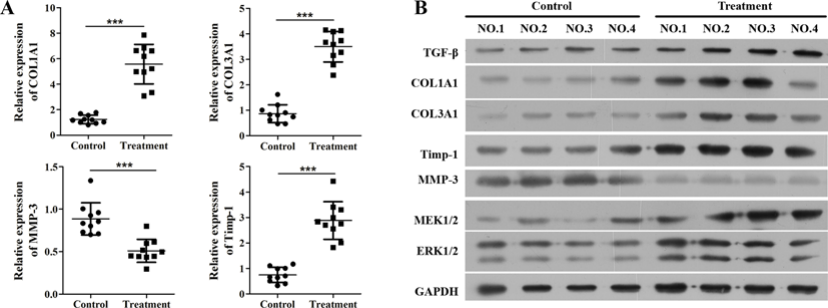
*D. genkwa* root extractives change extracellular matrix composition of patients’ granulation tissues. Expression levels of *COL1A1, COL3A1, Timp-1, MMP-3, TGF-β, MEK1/2* and *ERK1/2* in granulation tissues. (**A**) Expression levels of *COL1A1, COL3A1, TIMP-1* and *MMP-3* in 15 paired granulation tissues by qRT-PCR analysis. The levels of *COL1A1, COL3A1, Timp-1* were significantly up-regulated and the level of *MMP-3* was statistically reduced after the treatment of root extracts. ***P<0.05 compared with controls, *n*=10. (**B**) Western blot analysis of *COL1A1, COL3A1, Timp-1, MMP-3, TGF-β, MEK1/2* and *ERK1/2* protein expression in representative four paired granulation tissues. The expressions of *COL1A1, COL3A1, Timp-1, TGF-β, MEK1/2* and *ERK1/2* proteins were obviously elevated while the expression of *MMP-3* was remarkably decreased.

Comparison of curative effect between control group and *D. genkwa*-treated group revealed that *D. genkwa* significantly reduced the wound healing time (Table 1). To investigate the mechanism of *D. genkwa* on wound healing of AF patients, granulation tissues were obtained from the surface of wound for qRT-PCR and Western blot analysis. Since the level of collagen was evaluated for the phase of wound healing, *TIMP-1* protein and MMP were associated with the degradation of extracellular collagen, our data determined that as shown in Figure 1A, the expressions of *COL1A1, COL3A1, TIMP-1* mRNA were significantly up-regulated in granulation tissues, while the level of *MMP-3* was obviously down-regulated after *D. genkwa* treatment (*P*<0.001). Similar results were also observed in Western blot analysis (Figure 1B). Furthermore, we detected the expression of *TGF-β, MEK1/2* and *ERK1/2* using Western blot and found that they were all up-regulated after treatment with *D. genkwa* (Figure 1B).

**Table 1:** Comparation of curative effect between control group and *D. genkwa*-treated group

	Control	Treatment with *D. genkwa*
Gender		
Male	22	23
Female	8	7
Age (years)	36.7 ± 3.5	35.4 ± 2.1
Type		
High complexity	11	14
Low complexity	19	16
History (years)	3.1 ± 0.7	2.8 ± 0.8
Recent curative effect (recovery)	30	30
Wound healing time (d)	25.7 ± 3.4	18.1 ± 2.8*
Anal resting pressure after surgery (kPa)	10.37 ± 2.23	10.88 ± 1.96
Anal maximal contraction pressure after surgery (kPa)	13.41 ± 3.88	15.89 ± 3.22

*P<0.05, wound healing time in this group was significantly shorter as compared with control group.

**Figure 2:**
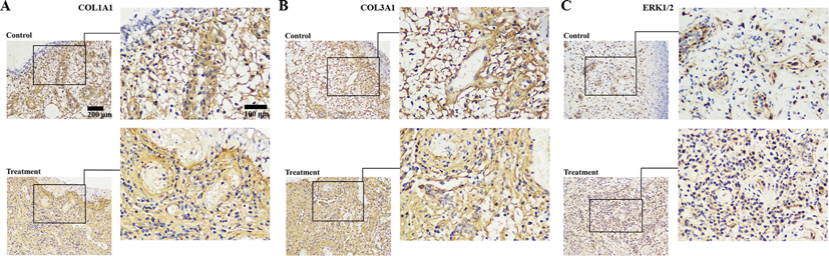
Analysis of COL1A1, COL3A1 and ERK1/2 proteins in granulation tissues by immunohistochemistry. *COL1A1* (**A**), *COL3A1* (**B**) and *ERK1/2* (**C**) expression were obviously increased in *D. genkwa*-treated granulation tissues, when compared with non-treated controls. Positive cells were stained brown. *ERK1/2-* positive cells were increased in *D. genkwa*-treated granulation tissues.

Because direct photos on private body parts were unavailable in practice, the immunohistochemical detection of collagen in the wound tissue was an indirect evaluation of wound healing instead. To verify this observation, we further examined the expression of *COL1A1, COL3A1* and *ERK1/2* in granulation samples by immunohistochemical staining. The results indicated that most of the granulation tissues showed positive staining of *COL1A1, COL3A1* and *ERK1/2* proteins after *D. genkwa* treatment compared with controls (Figure 2).

### *D. genkwa* root extractives favoured the proliferation of c-HSF cells

**Figure 3:**
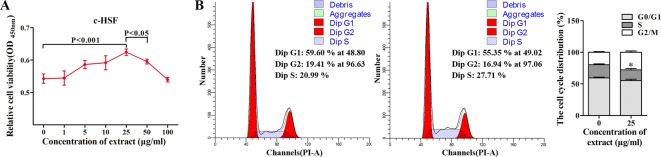
Effects of *D. genkwa* root extractives on c-HSFs. Effects of *D. genkwa* on the cell viability and cell cycle of c-HSFs, as determined by CCK-8 (**A**) and flow cytometry assay (**B**) respectively (*P<0.05, values are means of three replicates). The concentration of 25 μg/ml was optimal for the promotion of cell viability. The G_1_/S-phase of the cell cycle was decreased after the treatment of *D. genkwa*.

To further analyse the reparative process of *D. genkwa* root extractives in granulation tissues, c-HSF cells, as main constitutes of granulation tissues, were chosen to experiment *in vitro*. As shown in Figure 3A, 25 μg/ml *D. genkwa* root extractives caused remarkable promotion of c-HSF cell growth compared with the other concentration of *D. genkwa*. Additionally, no obvious side effect on the inflammation was found among patients with the treatment of the extract (25 μg/ml) though expressions of certain proteins were altered, compared with the patients with PBS. Based on this result, we chose 25 μg/ml *D. genkwa* root extractives as the optimal dose for the following assays. Flow cytometric analysis showed that treatment with 25 μg/ml extractives significantly decreased the arrest of cells at G_1_/S-phase of the cell cycle from 59.60%/20.99% in controls to 55.35%/27.71% in *D. genkwa* treatment group, indicating that a promoting role of *D. genkwa* on cell proliferation (Figure 3B).

### *D. genkwa* root induced the expression of collagen in fibroblasts

**Figure 4:**
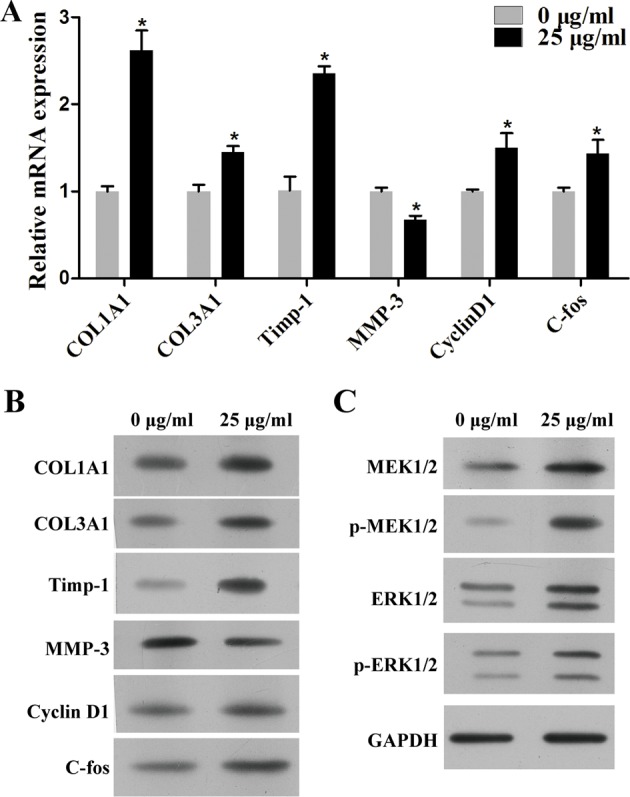
*D. genkwa* root extractives influence extracellular matrix composition of c-HSF cells througe inucing MEK/ERK signaling pathways. Mechanism study of *D. genkwa* in c-HSF cells. (**A**) qRT-PCR analysis of expression levels of *COL1A1, COL3A1, Timp-1, MMP-3, Cyclin D1* and *c-fos*. The levels of *COL1A1, COL3A1, Timp-1, Cyclin D1* and *c-fos* mRNAs were significantly increased while the level of *MMP-3* was down-regulated. *P<0.05 compared with controls. Values are means of three replicates. (**B**) The protein levels of *COL1A1, COL3A1, Timp-1, MMP-3, Cyclin D1* and *c-fos* in c-HSF cells after treatment with *D. genkwa.* (**C**) Western blotting analysis of *MEK1/2, p-MEK1/2, ERK1/2* and *p-ERK1/2*. The protein levels of *COL1A1, COL3A1, Timp-1, Cyclin D1, MEK1/2, p-MEK1/2, ERK1/2* and *p-ERK1/2* were obviously up-regulated while *MMP-3* was down-regulated after the treatment of extracts.

To examine the regulatory mechanism of *D. genkwa*, multiple signalling pathways were analysed in c-HSF cells. Consistent with the results of tissues, 25 μg/ml *D. genkwa* root extractives obviously elevated the expression of *COL1A1, COL3A1, TIMP-1, Cyclin D1* and *c-fos* mRNAs, whereas reduced the expression of *MMP-3* mRNA (Figure 4A, *P*<0.05). In addition, we also detected the protein levels of these molecules using Western blot. As shown in Figure 4B, except *MMP-3*, the expression of *COL1A1, COL3A1, TIMP-1, Cyclin D1* and *c-fos* mRNA levels were obviously up-regulated in c-HSF cells after treatment with 25 μg/ml *D. genkwa* root extractives. As expected, 25 μg/ml *D. genkwa* root extractives notably promoted the activation of *MEK/ERK* pathway by up-regulating the phosphorylation of *MEK1/2* and *ERK1/2*, indicating *D. genkwa* root extractives affected cell proliferation possibly via the activated MEK/ERK pathway (Figure 4C).

### *D. genkwa* root extractives could protect HSF cells against the damage by TNF-α

**Figure 5:**
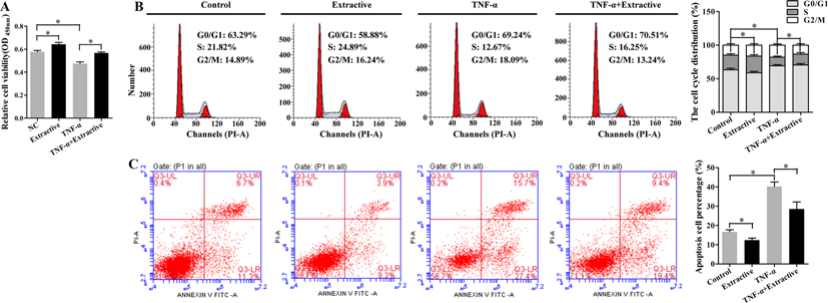
*D. genkwa* root extractives protect cells against *TNF-α* damage. Effects of *D. genkwa* on HSF cells damaged by *TNF-α*. The cell viability (**A**), cell cycle (**B**) and apoptosis (**C**) of HSF cells treated with *D. genkwa* extractives, *TNF-α, TNF-α* + *D. genkwa* extractives respectively using CCK-8 assay and flow cytometry assay (*P<0.05 compared with control. Values are means of three replicates). The root extractives remarkably alleviated the suppression of cell proliferation, decreased cell early and late apoptosis by *TNF-α*.

Next we investigated the effects of *D. genkwa* on impaired HSF cells. We constructed damaged HSF cell model by treating cells with *TNF-α*. As shown in Figure 5A, *TNF-α*, as a pro-inflammatory cytokine significantly reduced the cell viability compared with normal HSF cells (*P*<0.05). Interestingly, *D. genkwa* root extractives obviously elevated the relative cell viability of impaired HSF cells, suggesting that *D. genkwa* root extractives might protect impaired cells against *TNF-α* damage.

### *D. genkwa* extractives facilitated cell proliferation and suppressed cell apoptosis induced by TNF-α

Subsequently, we determined whether *D. genkwa* root extractives affected cell-cycle progression and apoptosis in impaired HSF cells. As shown in Figure 5B, *TNF-α* remarkably induced cell-cycle arrest at S-phase from 63.29%/21.82% in control group to 69.24%/12.67% in *TNF-α* group, indicating a negative effect of *TNF-α* on cell proliferation, but this cell-cycle arrest was notably alleviated by addition of *D. genkwa* extractives. Consistent with the result of cell cycle, *TNF-α* induced early apoptosis of cell from 11.3 to 27.4% and late apoptosis from 6.7 to 15.7%. Surprisingly, *D. genkwa* root extractives dramatically decreased cell early apoptosis from 27.4 to 19.4% and late apoptosis from 15.7 to 9.4% in impaired HSF cells (Figure 5C).

### *D. genkwa* root extractives suppressed TNF-α induced wounding by regulating expression of molecules associated with healing

**Figure 6:**
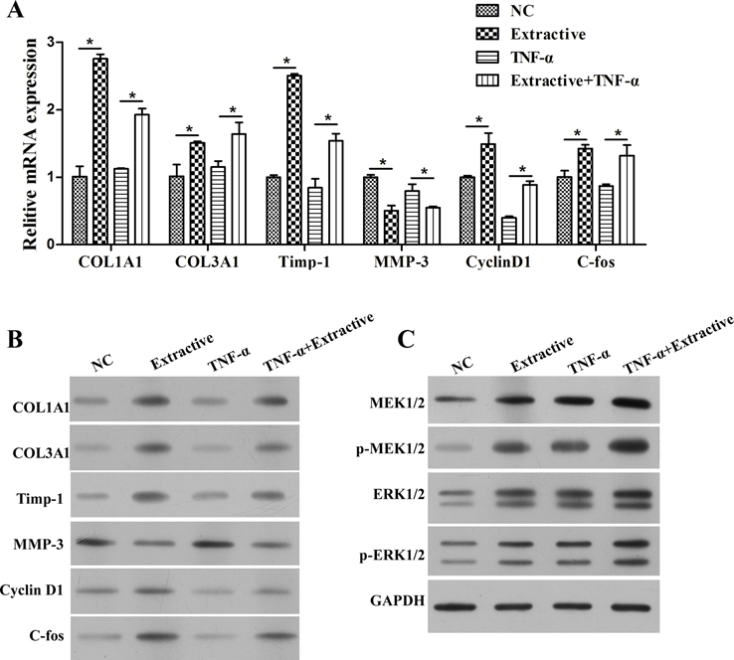
Mechanism study of *D. genkwa* extractives in HSF cells after treatment with TNF-α. (**A**) qRT-PCR analysis of expression levels of *COL1A1, COL3A1, Timp-1, MMP-3, Cyclin D1* and *c-fos*, *P<0.05 compared with controls or TNF-α group. Values are means of three replicates. (**B**) The protein levels of *COL1A1, COL3A1, Timp-1, MMP-3, Cyclin D1* and *c-fos* in HSF cells. (**C**) Western blotting analysis of *MEK1/2, p-MEK1/2, ERK1/2* and *p-ERK1/2*. The root extractives obviously increased the levels of *COL1A1, COL3A1, Timp-1, Cyclin D1, c-fos, MEK1/2, p-MEK1/2, ERK1/2* and *p-ERK1/2* and decreased *MMP-3* level affected by *TNF-α*.

To further detect the molecular mechanism of *D. genkwa* on proliferation of impaired HSF cells, we also measured the expression of extracellular matrix (ECM) and *MEK/ERK* signalling pathways. As shown in Figure 6A, soluble collagen (*COL1A1* and *COL3A1*), *Timp-1, Cyclin D1* and *c-fos* were all significantly down-regulated in HSF cells induced by *TNF-α*, but obviously up-regulated under the existence of *D. genkwa* root extractives (*P*<0.05). Similar results were verified using Western blot analysis (Figure 6B). In addition, the activation of *MEK/ERK* signalling pathways further demonstrated the protective roles of *D. genkwa* root extractives for the repair of *TNF-α*-damaged HSF cells, as revealed by the up-regulation of *p-MEK1/2* and *p-ERK1/2* (Figure 6C).

## Discussion

In our study, thread-drawing method was used for the therapy of AF. The operation line in the experimental group was presoaked in the roots extract of* D. genkwa* (boiled) and the root extract was also applied outside the wound site. The operation line boiled with the extract followed the way of traditional Chinese medicine. *D. genkwa* root has been reported to possess antitumour and anti-inflammatory activities, but its effects on wound healing remain largely unknown. The key finding of the present study was the root extractives of *D. genkwa* could promote wounding healing by accelerating granulation tissue formation and facilitating fibroblast cell proliferation via multiple signalling pathways. These results suggested that *D. genkwa* root functioned as a positive factor during the wound healing process.

Further investigation indicated that the root extractives of *D. genkwa* elevated the expressions of type I collagen (*COL1A1* and *COL3A1*)*, TIMP-1, c-fos* and *Cyclin D1* at mRNA and protein levels in granulation tissues and fibroblast cells. As we all know, ECM was involved in directing epithelial cell functions [16]. As a major structural component of ECM, type I collagen played an important role in remodelling ECM microenvironment [17,18]. In our results, up-regulation of *COL1A1* and *COL3A1* showed *D. genkwa* might enhance the function of ECM by regulating type I collagen. Activating protein-1 (*AP-1*) mainly participated in JNK stress pathway via the regulation of cell growth, differentiation and apoptosis [19]. *c-fos* has been identified as a major component of *AP-1* complex and implicated in signal transduction, cell proliferation and angiogenesis [20,21]. Additionally, mouse fibroblast cell lines deficient in *c-fos* was found to be more sensitive to radiation by which cell apoptosis was intensified [22,23]. We also observed *Cyclin D1* was up-regulated by the root extractives of *D. genkwa*, which was closely associated with cell cycle at G_0_/G_1_-phase [24] in fibroblast cells. As evidence showed that the induction of G_1_/S-phase arrest was engaged with cell apoptosis, our data validated that *D. genkwa* promoted the cell proliferation via inhibiting the arrests of cells at G_1_ /S-phase. It has been demonstrated that *MMPs* belonged to a family of metal-dependent proteolytic enzymes that degraded the ECM components and participated in bone remodelling, wound healing and apoptosis [25]. *Timps* were specific inhibitors of *MMPs* and there was a balance among them [26]. In agreement with the evidence, our result indicated that *D. genkwa* significantly elevated the expression of *Timp-1*, but reduced the expression of *MMP-3*, which had a significant effect on wound healing.

We found *D. genkwa* elevated phosphorylation levels of *MEK/ERK*. Previous study showed that the classic *MEK/ERK* pathway was a key signal transduction component of cell proliferation in many cells, which contains a cascade of protein kinases: *MEK* and *ERK* [27]. Activation of *MEK/ERK* signalling pathway has been shown to contribute to cell growth, invasion and EMT [28,29]. These data further support our results that activation of *MEK/ERK* pathway was required for *D. genkwa* stimulated cell proliferation in granulation tissues as well as the growth of fibroblast cells. Interestingly, the total expressions of *MEK* and *ERK* were also elevated. For we cannot exclude difference of the total *MEK/ERK* of the clinical samples detected by WB, we will further analyse the effective components of the extract, and it is more appropriate to determine the mechanism of activation of *MEK/ERK* pathway with the monomer. However, in the present study, we additionally built damaged cell model of HSF and also determined the effects of *D. genkwa* on these damaged cells. Consistently, we found *D. genkwa* could effectively protect cells against the damage of *TNF-α* and reverse the inhibition of cell growth and induction of apoptosis, via activating *MEK/ERK* signalling pathways and ECM components. In summary, we demonstrated for the first time that *D. genkwa* root played a crucial role in wound healing via protecting granulation tissues and fibroblast cells. We also found the function of *D. genkwa* root in wound healing had a close relation with ECM components and *MEK/ERK* signalling pathway. The extracts induced the expression of *Timp-1* protein and inhibited the degradation of extracellular collagen by MMP. The deposition of collagen itself was also considered as the manifestation of wound healing, along with the activation of *MEK/ERK* signalling pathway in the promotion of skin fibroblast proliferation, which together facilitated the fibrosis of wound healing and led to wound closure.

As the difference of treatment between AF wound and ordinary wound was the source of infection, the wound healing difficulty of AF was mainly due to contamination of *Escherichia coli* in faeces. However, the specific bacteriostatic effect of the extracts needs to be further investigated. Besides, the clinical value of these findings requires to be evaluated before *D. genkwa* root was applied to shorten the period of wound healing in patients with AFs. The exact mechanism of *D. genkwa* on the regulation of *COL1A1* and *COL3A1* needs further studies such as using k/o experiments to be clarified. The application of *D. genkwa* in wound healing still requires the official approval such as Food and Drug Adminisration (FDA).

Taken together, our preliminary data demonstrated the therapeutic effect of *D. genkwa* on AF and showed that root extractive from *D. genkwa* benefited the wound healing through up-regulation of collagen genes in HSFs.

## Abbreviations

AF: anal fistula
AP-1: activating protein-1
CCK-8: cell count kit-8
c-HSF: cheloid human skin fibroblast
COL1A1: collagen type I alpha 1 chain
COL3A1: collagen type III alpha 1 chain
ECM: extracellular matrix
ERK: extracellular regulated protein kinases
HRP: horseradish peroxidase
HSF: human skin fibroblast
MEK: mitogen-activated protein kinase kinase
MMP: matrix metalloproteinase
PI: propidium iodide
qRT-PCR: quantitative real-time PCR
TNF: tumour necrosis factor
TGF-β: transforming growth factor beta
Timp: tissue inhibitor of metalloproteases
